# Screening the Digital Skills of Patients in Geriatric Rehabilitation: Multicenter Cross-Sectional Study

**DOI:** 10.2196/84425

**Published:** 2026-04-02

**Authors:** Michael BG Zonneveld, Margriet C Pol, Marise J Kasteleyn, Wilco P Achterberg, Eléonore F van Dam van Isselt

**Affiliations:** 1 Department of Public Health and Primary Care Leiden University Medical Center Leiden The Netherlands; 2 University Network for the Care Sector Zuid-Holland Leiden University Medical Center Leiden The Netherlands; 3 Department of Medicine for Older People Amsterdam Public Health Research Institute Amsterdam University Medical Center Amsterdam The Netherlands; 4 Research Group Occupational Therapy-Technology and Participation Faculty of Health Amsterdam University of Applied Sciences Amsterdam The Netherlands; 5 National eHealth Living Lab (NeLL) Leiden The Netherlands

**Keywords:** older adults, telehealth, telerehabilitation, rehabilitation, digital health, digital proficiency, assessment

## Abstract

**Background:**

Digitalization in geriatric rehabilitation presents unique challenges, making it essential to align eHealth solutions with patients’ digital skills. The Quickscan Digital Skills (QDS) is a tool designed to help health care professionals match eHealth interventions to individual skill levels.

**Objective:**

This study aimed to explore the applicability of QDS by comparing it to self-reported digital skills and to gain insight into the digital skills of patients in geriatric rehabilitation.

**Methods:**

In this multicenter cross-sectional study, participants from 13 geriatric rehabilitation centers in the Netherlands completed a survey, including demographic questions, QDS, and a numeric rating scale (NRS) for self-reported digital skills. Participants were categorized into 3 skill levels (beginner, intermediate, and experienced) based on the cutoff points in QDS scores. Cutoff points were predetermined, guided by the information provided on QDS. Descriptive statistics for median age and frequencies for skill levels were calculated. Comparative analysis using a Kruskal-Wallis test assessed differences between QDS and NRS within these groups, and Spearman rank-order correlation examined the relationship between the two measures. To gain more insight into the different skill levels between groups, data were visualized and associations among age, gender, and digital skill levels were examined using ordinal logistic regression analysis.

**Results:**

A total of 463 patients (median age 78, IQR 12 years; 282/463, 60.9% female) participated in this study. Based on QDS scores, 42.1% (195/463) were classified as beginners, 19.4% (90/463) as intermediates, and 38.4% (178/463) as experienced users. A moderate positive correlation was found between QDS and NRS scores. Digital skills generally declined with age: 69.8% (37/53) of participants younger than 65 years were experienced users compared to only 13.2% (5/38) of those older than 91 years. A logistic regression analysis showed that increasing age was significantly associated with lower digital skill levels (odds ratio 0.93, 95% CI 0.92-0.95; *P*<.001). The association between age and digital skills does not differ between males and females.

**Conclusions:**

This study suggests that QDS is a promising and practical screening tool for assessing digital skills in patients in geriatric rehabilitation. Self-reported digital skills with an NRS do not capture the differentiation in the assessed abilities by QDS. QDS could be a practical tool for identifying digital skill levels in patients in geriatric rehabilitation and can support more personalized eHealth implementation. Further research should explore the parametric properties of QDS and how the scores relate to actual eHealth use.

## Introduction

eHealth is becoming increasingly important in geriatric rehabilitation and has been found to be effective as long as it aligns with the digital skills and needs of the patient [1]. Within geriatric rehabilitation, digitalization presents unique challenges, particularly given the diversity in life experiences and backgrounds of older adults [2]. These challenges are compounded by age-related barriers such as impaired vision, dexterity issues that result in difficulties with touch screens, or cognitive limitations [3,4].

The World Health Organization has emphasized the importance of eHealth aligning with the users’ digital health literacy and skills in its global strategy on digital health [5]. Digital health literacy is defined as the ability to seek, find, understand, and appraise health information from electronic sources and apply the knowledge gained to address or solve a health problem. When there is a mismatch between digital health literacy and the skills of the users and the rate at which society is digitalized, existing health inequalities (eg, access to the internet and the skills to use the internet) can increase [6]. As a result of the digitalization of society, people could end up receiving inadequate care, which could increase health inequalities even more.

Despite the Netherlands having a notably high percentage of daily internet users compared to other European countries (91.4% among adults aged 65-75 years), it remains unclear what their digital health literacy and skills are. Usage among those aged 75 years and older declines to 66.3%, which is a major demographic in geriatric rehabilitation [7]. Promoting a positive culture around digital technology use is essential to ensure older adults maintain engagement, even when their digital health literacy is limited [8,9].

eHealth inequalities experienced by older adults can be exacerbated by societal and self-directed ageism [3,8]. Self-ageism refers to internalized age-based discrimination [10,11], which may lead older adults to perceive themselves as less digitally capable and therefore avoid learning new digital skills [3]. By avoiding these negative experiences, older adults decrease their digital technology use, which poses extra challenges to the digitalization of health care systems that focus on treating older adults. Societal ageism is present among health care professionals who tend to assume older adults are less digitally skilled and, therefore, exclude the option of eHealth from the start [12-14]. Therefore, the assessment of the fit with the individual patient’s skill level, needs, and physical capabilities cannot solely depend on the judgment of the health care professional. Similarly, self-assessment by older adults introduces its own limitations, as individuals may inaccurately estimate their abilities due to internalized biases or limited reference frameworks [15]. Therefore, an assessment of the digital skills of a patient at the start of geriatric rehabilitation could aid health care professionals in their decision to offer a care pathway utilizing eHealth effectively [16].

To our knowledge, there is no digital skills assessment specifically for geriatric rehabilitation. Therefore, there is a need to look into related fields such as hospital care. Recently, a scoping review on digital health literacy in a hospital setting found limited results on the inclusion of digital skills within these assessments, with only 8 out of 44 assessments having any form of digital skills included [16]. One of the assessments, the eHealth Literacy Questionnaire, has been studied and validated but is intended to be used by policymakers, eHealth developers, and researchers [17]. A more performance-based instrument is the Digital Literacy Instrument, which consists of 21 self-report items with a 4-point Likert scale, which is still quite extensive with 21 items [18]. The shortest assessment is the eHealth Literacy Scale (eHEALS), which includes 8 items answered on a 5-point Likert scale [19]. However, the eHEALS has been previously discussed as being outdated in its questions since its development in 2006 [20,21].

Pharos, the Dutch Centre of Expertise on Health Disparities, developed the Quickscan Digital Skills (QDS) [22]. QDS was developed as a practical digital skills assessment tool for health care professionals, consisting of 6 items. What sets QDS apart from the other assessments is that, in its conclusion, it tells you what kind of support is necessary when eHealth is used and which types of eHealth might fit [22]. However, QDS has not yet been scientifically validated and is not being used regularly in geriatric rehabilitation.

This study has two main objectives: first, to explore the applicability of QDS by comparing it to self-reported digital skills, and second, to examine how digital skills vary among patients in geriatric rehabilitation based on demographic factors.

## Methods

This multicenter cross-sectional study used a survey. Data were collected from October 2023 to April 2024 by trained occupational therapy students in a face-to-face, interview-like setting.

### Patient Recruitment

Patients were recruited from 13 geriatric rehabilitation centers in the Netherlands, both urban and rural. All the participating centers were affiliated with a university network (UNC ZH, UNO Amsterdam, UKON [23-25]), and they were approached through these networks for participation. Patients could also be excluded during data collection due to insufficient physical or cognitive capacity. Reasons for exclusion were registered.

### Ethical Considerations

The non-Dutch Medical Research Involving Human Subjects Act (WMO) review board of the Leiden University Medical Center (one of the institutional review boards) declared this study outside the scope of the Dutch Medical Research Involving Human Subjects Act and issued a certificate of no objection (reference 23-3072) [26]. All patients in the geriatric rehabilitation ward of the participating rehabilitation centers were eligible for inclusion unless the staff excluded them beforehand if they were known to receive palliative care or did not speak Dutch. Informed consent was obtained verbally and registered before continuing with the survey. All patients had the option to opt out of the study without any consequences. All data were collected anonymously and could not be traced back to the participant. No compensation was given to participants.

### Data Collection

#### Patient Characteristics

Demographic data, including age, gender, and rehabilitation diagnosis, were collected. Rehabilitation diagnoses were categorized into 4 nationally recognized groups: stroke, orthopedics and trauma, amputation, and others. The rehabilitation center where the patient resided was also recorded. For comparison with national data, age was categorized in 5-year increments. Participants were asked to rate their digital skills on a numeric rating scale (NRS), with scores ranging from 1 to 10, with higher scores indicating higher digital skills. To make sure participants understood the NRS and what they were rating, it was pilot tested. The NRS was accompanied by the explanation that 1 means no digital skills and 10 means high digital skills. All data were collected in a face-to-face interview-like setting by occupational therapy students who received a thorough explanation of the questions and data collection system. All data were entered directly and anonymously into Castor EDC [27].

#### QDS Tool

The digital skills were assessed using QDS [22]. It was developed as a practical assessment for health care professionals to decide what kind of support is needed when eHealth is used according to the patient's skill level. The QDS contains 6 questions (Table 1), which can all be answered with No, With help, or Yes. The QDS guides assessors in interpreting the digital skill level of the participant based on their responses: if the participant answered mostly “No” and “With help,” they are seen as not digitally skilled or beginners who need help with the use of the internet and applications. If they answered equal amounts of “With help” and “Yes,” the conclusion was drawn that they are of intermediate skill level and that these people need help sometimes. When they answered mostly “Yes,” they were classified as experienced: they can largely use the internet and applications independently. In this study, the responses were quantified to allow for standardized analysis. The following scoring system was applied: No=0, With help=1, and Yes=2. Adding the scores on all the answers results in a total Quickscan score ranging from 0 to 12. To then determine the digital skill level, cutoff points were set at “not digitally skilled or beginner = ≤6,” “intermediate = 7-9,” and “experienced = ≥10.” These scores were based on the interpretation guideline provided by QDS.

**Table 1:** The translated questions of the Pharos Quickscan Digital Skills tool.

	No	With help	Yes
Do you have a computer, phone, tablet with internet?			
Do you ever look for information (about health and diseases) on the internet?			
Are you using email so I can send you a link?			
Do you ever use an app?			
Can you download an app yourself?			
Do you use your DigiD to view your patient data, for example?			

### Data Analysis

Following data cleaning, descriptive analyses were performed on the demographics by using mean (SD) for normally distributed data, median (IQR) for nonnormally distributed data, and frequencies with percentages for categorical variables. Several comparative analyses were performed to determine if there were significant differences between the digital skill level and NRS. First, the distribution of the total QDS scores in relation to NRS was visualized with a bubble plot. Second, a Kruskal Wallis test was conducted to compare NRS with the 3 digital skill levels, and post-hoc analyses, using Dunn's (1964) procedure with a Bonferroni correction for multiple comparisons, were applied when necessary. Third, Spearman rank-order correlation was used to explore the association between the categorical digital skill level and the NRS scores in greater detail. To gain more insight into the digital skills of the participants, differences between groups based on gender, age, and diagnosis groups were visualized and described with frequencies and percentages. Associations between demographic characteristics (age and gender) and digital skill levels were examined using ordinal logistic regression analysis. Model assumptions, including the proportional odds assumption, were evaluated using the test of parallel lines.

## Results

### Study Participants

**Figure 1:**
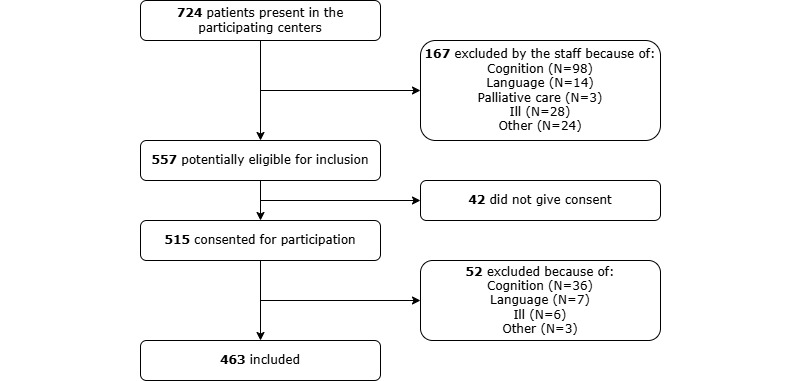
Flowchart of the inclusion and exclusion of participants.

A total of 724 patients from 13 distinct geriatric rehabilitation centers were approached for participation. Of these, 167 individuals were excluded by the staff due to various reasons, including cognitive impairments, language barriers, palliative care, illness, or other factors. Among the remaining 557 eligible patients, 42 declined to provide consent, resulting in 515 consenting participants. During data collection, 52 patients were excluded by the researchers because they noticed a language barrier, a lack of understanding of the questions due to cognitive problems, illness, or other reasons. Consequently, the final sample comprised 463 participants, yielding an inclusion rate of 64% (463/724). Detailed participant inclusion and exclusion are shown in Figure 1. 

### Demographics

The sample was predominantly female (282/463, 60.9%), with a median age of 78 (IQR 12) years. Most participants were aged between 76 and 80 years (116/462, 25.1%), while the smallest group comprised those aged 91 years and older (38/462, 8.2%). The most common diagnosis for rehabilitation was orthopedics and trauma (200/463, 43.2%), followed by other diagnoses (144/463, 31.1%) and stroke (77/463, 16.6%). Overall, digital skill levels as measured with QDS were nondigitally skilled or beginners (195/463, 42.1%), intermediate (90/463, 19.4%), and experienced (178/463, 38.4%) users. For a complete overview of the demographics, see Table 2.

**Table 2:** Overall demographics of the participants classified by digital skill levels.

			Total, n (%)		Nondigitally skilled and beginners, n (%)		Intermediate, n (%)		Experienced, n (%)
Overall			463 (100)		195 (42.1)		90 (19.4)		178 (38.4)
**Sex**									
	Male	181 (39.1)		67 (37)		33 (18.2)		81 (44.8)	
	Female	282 (60.9)		128 (45.4)		57 (20.2)		97 (34.4)	
**Age (y)**									
	<65	53 (11.5)		8 (15.1)		8 (15.1)		37 (69.8)	
	66-70	47 (10.2)		14 (29.8)		14 (29.8)		19 (40.4)	
	71-75	78 (16.9)		25 (32.1)		17 (21.8)		36 (46.2)	
	76-80	116 (25.1)		48 (41.4)		19 (21.1)		49 (42.2)	
	81-85	79 (17.1)		41 (51.9)		15 (19)		23 (29.1)	
	86-90	51 (11)		31 (60.8)		11 (21.6)		9 (17.6)	
	>91	38 (8.2)		27 (71.1)		6 (15.8)		5 (13.2)	
	Missing	1 (0.002)		—^a^		—		—	
**Diagnosis**									
	Stroke	77 (16.6)		30 (39)		18 (23.4)		29 (37.7)	
	Orthopedics and trauma	200 (43.2)		90 (45)		37 (18.5)		73 (36.5)	
	Amputation	18 (3.9)		10 (55.6)		0 (0)		8 (44.4)	
	Other	144 (31.1)		52 (36.1)		27 (18.8)		65 (45.1)	
	Unknown	24 (5.2)		13 (54.2)		8 (33.3)		3 (12.5)	

^a^Not applicable.

### Applicability of QDS vs NRS

**Figure 2:**
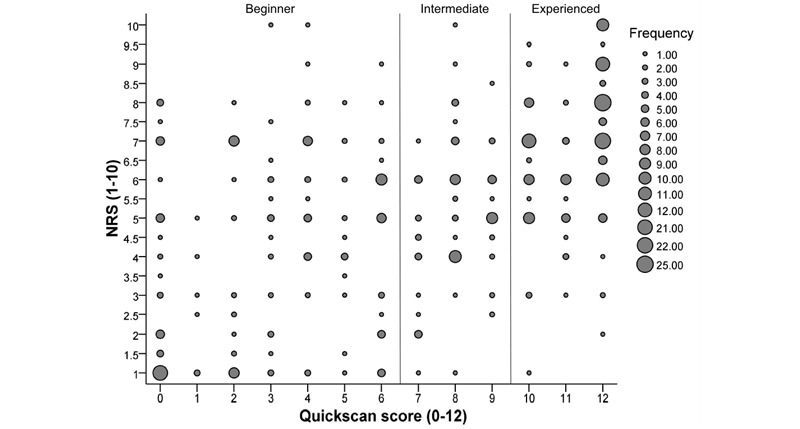
Bubble plot of the Quickscan digital skills scores vs numeric rating scale (NRS) scores and their frequencies.

A bubble plot (Figure 2) illustrates the distribution and frequency of total QDS scores in relation to the NRS values. The bubble plot shows a considerable variation in each digital skill group with various frequencies. The mean ranks of NRS scores were statistically significantly different between groups (*χ*^2^_2_=126.8; *P*<.001). Post-hoc analyses revealed statistically significant differences in mean rank NRS scores between the beginners and the experienced groups (*P*<.001) and the intermediates and the experienced (*P*<.001) but not between the beginners and intermediates (*P*=.045). There was a statistically significant, moderate positive correlation between the total QDS and NRS (*r*_s_(460)=0.554, 95% CI 0.485-0.616; *P*<.001) scores.

### Insight in Digital Skills

**Figure 3:**
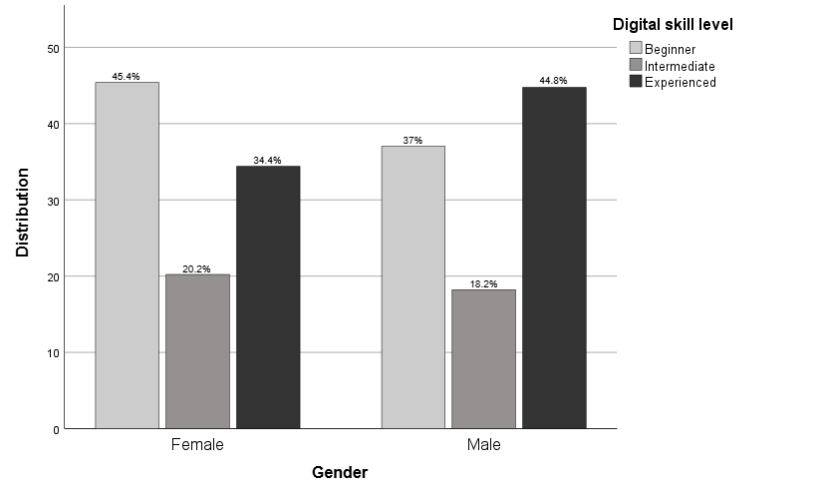
Visualization of skill level distribution by gender.

**Figure 4:**
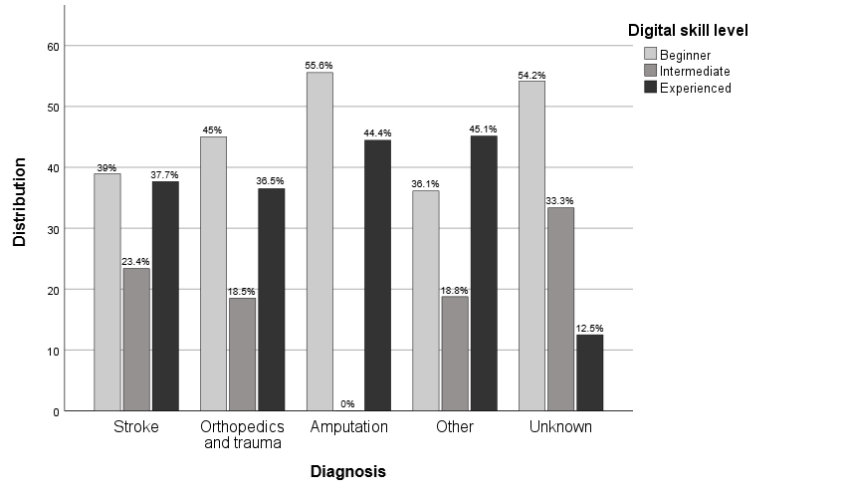
Visualization of skill level distribution by diagnosis.

Skill levels differed between male and female participants. Most male participants were experienced users (81/181, 44.8%), whereas the largest proportion of female participants were nondigitally skilled or beginners (128/282, 45.4%) (Figure 3). There was a similar distribution between the diagnosis groups and the overall group, except for the amputation and the other groups, which both had a higher percentage of beginners (10/18, 55.6% and 13/24, 54.2%, respectively) as can be seen in Figure 4.

**Figure 5:**
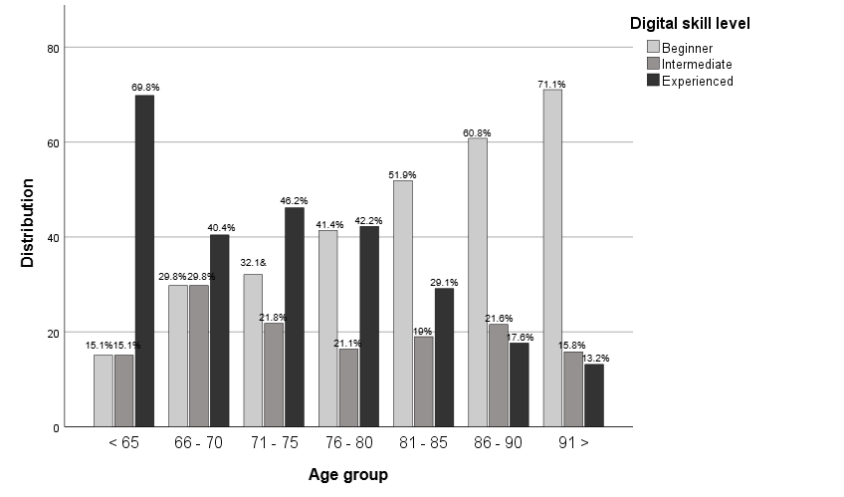
Visualization of skill level distribution by age.

The digital skill level for each age group showed a decrease in skill level with age, with 69.8% (37/53) of those younger than 65 years in the experienced group compared to 13.2% (5/38) in those who older than 91 years (Figure 5). Conversely, the proportion of nondigitally skilled or beginners steadily increased with age, from 15.1% (8/53) in the <65 years age group toward 71.1% (27/38) in the >91 years age group. The proportions of the intermediate users remained relatively stable at approximately 20% across all age groups.

Due to the observed differences in gender and age, an ordinal logistic regression analysis was done to examine whether gender differences in digital skill level were independent of age. Increasing age was significantly associated with lower digital skill levels (odds ratio 0.93, 95% CI 0.92-0.95; *P*<.001). After adjusting for age, gender was no longer significantly associated with digital skill level (*P*=.15). Furthermore, the age × gender interaction term was not significant (*P*=.49), indicating that the association between age and digital skills does not differ between males and females.

## Discussion

### Principal Findings

This study aimed to explore the applicability of QDS as a tool for screening digital skills in patients in geriatric rehabilitation by comparing it with a self-reported NRS and to gain insight into the digital skill levels of patients undergoing geriatric rehabilitation.

The study demonstrated notable discrepancies between QDS and NRS, highlighting the practical use of QDS as an objective screening tool. QDS differentiated patients into 3 distinct skill categories: nondigitally skilled or beginner, intermediate, and experienced. Notably, the majority of patients were classified as having intermediate or experienced digital skill levels. Interestingly, while increasing age was significantly associated with a decline in digital skills, the data revealed exceptions with some individuals aged older than 91 years demonstrating high skill levels and others younger than 65 years showing low levels of digital competencies. This shows that assuming low digital skills solely based on age is not accurate.

The moderate correlation between QDS and NRS, combined with clear discrepancies in individual scores, highlights important limitations of self-assessment. Some patients scored themselves highly on NRS, yet were classified as having low digital skills on QDS and vice versa. These inconsistencies may reflect the subjective nature of a self-evaluation with it being difficult to score oneself if not familiar with the topic at hand [28]. This results in a different reference framework used by each person, and though unknown to the assessor, will influence the scores given [29]. For example, participants may have compared themselves to either highly skilled individuals or others with minimal digital exposure. The reference frameworks could be influenced by the person’s self-perception and therefore be a result of self-directed ageism [11].

These subjective evaluations suggest that while NRS may capture participants’ motivation and openness toward eHealth, QDS offers a more structured and objective assessment of digital skills. This distinction is critical, as prior research has shown that older adults’ self-reported eHealth literacy often does not align with their actual ability to use digital tools effectively [30]. This could again be related to personal beliefs and self-perception, the continuum of digital literacy, and the contextual barriers and facilitators to technology adoption [31].

Although QDS is not yet a validated or standardized instrument, it provides a replicable and systematic approach for assessing digital skills in clinical practice. Its quantified design minimizes interpretative variability during administration, offering a key advantage over subjective self-rating scales. While this study was not intended to establish the psychometric properties of QDS, the findings offer preliminary support for its practical utility. This underscores the importance of having reliable tools to identify individuals who may be less likely to benefit from digital health interventions [32].

### Strengths and Limitations

The first strength of this study is its sample size and how it compares to national and international data, showing its representativeness of the population. Our study sample aligns with national data on geriatric rehabilitation, with 60.9% (282/463) females in this study compared to 59.6% (36,015/60,450) nationally [33]. Diagnoses such as stroke showed identical prevalence, while orthopedics and trauma were similar. For age, it seems the population in this study was a bit younger, with a median age of 78 years compared to the estimated European mean age of 80 years [34].

Another strength lies in the quantification of QDS, which minimizes interpretive variability during data collection, even though the reliability of QDS has not been studied [35]. Nonetheless, quantifying QDS was not without limitations. Participants who answered 4 questions with “Yes,” one “With help,” and one “No” ended up being classified as intermediate skill level. The interpretation guideline by Pharos mentions someone should be experienced if they answer mostly “Yes” [22]. For this study, the research group argued that anyone who answers a question with “No” should not be classified as experienced and was therefore used as the upper threshold for intermediate classification. There was also no possibility of comparing QDS with a gold standard, since there currently is none [16].

A limitation of this study is that QDS has not yet been formally validated. Although the instrument was developed as a practical screening tool, its psychometric properties have not been formally evaluated. This may have resulted in measurement imprecision or misclassification of digital skill levels, particularly within intermediate categories. Nevertheless, this study provides preliminary empirical insight into the clinical applicability of QDS and highlights the need for formal validation studies in geriatric rehabilitation.

Despite the relatively large sample, another limitation is the fact that some patients either did not consent to participation or were excluded in advance by the health care professionals. The potential impact of these nonparticipants remains unknown and has been a known problem in eHealth research [36-38]. In particular, the applicability of QDS for those who were excluded by the staff based on cognition remains unknown. An underrepresentation of people with cognitive problems could result in overlooking flaws in the questions of QDS that could not be found with their participating counterparts.

### Implications for Geriatric Rehabilitation and eHealth

Our findings suggest that QDS may serve as a valuable tool for tailoring eHealth interventions to older adults’ skill levels. It offers health care professionals practical insight into patients’ digital abilities and can help guide decisions about the level of support required. QDS could therefore be an eHealth readiness indicator for the patient that helps with shared decision-making. Due to its observed short administration time, integration into current workflows seems feasible. However, QDS was not originally developed for the geriatric rehabilitation context. Future studies should evaluate whether QDS validly measures digital skills and clarify how scores translate into actionable care decisions before widespread adoption in clinical practice.

### Conclusions

This study suggests that QDS is a promising and practical screening tool for assessing digital skills in patients in geriatric rehabilitation. QDS seems to be applicable as a screening tool when compared to self-reported digital skills on an NRS, since it revealed a differentiation in skill levels not captured by self-reported NRS scores. Even though QDS is not a gold standard, it could provide practical guidance to health care professionals when they want to use eHealth with a patient. QDS showed a differentiation into 3 skill levels and gives examples of what to offer for each skill level and what kind of help these people would need. However, the examples are not specifically designed for patients in geriatric rehabilitation, underscoring the need for further research to validate its practical applications within this field. Furthermore, future studies should focus on assessing the digital skills of patients in geriatric rehabilitation and on linking the results of QDS with practical skills and help needed with eHealth use in geriatric rehabilitation.

## Data Availability

Data can be made available upon request to the corresponding author.

## Abbreviations

eHEALS: eHealth literacy scale
NRS: numeric rating scale
QDS: Quickscan digital skills
